# Population genetic structure of the land snail *Camaena cicatricosa* (Stylommatophora, Camaenidae) in China inferred from mitochondrial genes and ITS2 sequences

**DOI:** 10.1038/s41598-017-15758-y

**Published:** 2017-11-15

**Authors:** Weichuan Zhou, Haifang Yang, Hongli Ding, Shanping Yang, Junhong Lin, Pei Wang

**Affiliations:** 1 Key Laboratory of Molluscan Quarantine and Identification of AQSIQ, Fujian Entry-Exit Inspection & Quarantine Bureau, Fuzhou, Fujian, 350001 China; 2 National Wetland Museum of China, Hangzhou, Zhejiang, 310013 China; 3 College of Plant Protection, Fujian Agriculture and Forestry University, Fuzhou, Fujian, 350002 China

## Abstract

The phylogeographic structure of the land snail *Camaena cicatricosa* was analyzed in this study based on mitochondrial gene (*COI* and *16srRNA*, mt DNA) and internal transcribed spacer (ITS2) sequences in 347 individuals. This snail is the vector of the zoonotic food-borne parasite *Angiostrongylus cantonensis* and one of the main harmful snails distributed exclusively in China. The results revealed significant fixation indices of genetic differentiation and high gene flow between most populations except several populations. An isolation-by-distance test showed no significant correlation between genetic distance and geographical distance among *C. cicatricosa* populations, which suggested that gene flow was not restricted by distance. The levels of haplotype and nucleotide diversity of *C. cicatricosa* were generally high, except those in some special populations, according to the mt DNA and ITS2 data. Furthermore, the phylogenetic trees and asteroid networks of haplotypes indicated nonobvious genetic structure, the same as results got based on the synonymous and non synonymous sites of 347 sequences of the *COI* gene. All lines of evidence indicated that climatic changes and geographical and human barriers do not substantially affect the current population structure and distribution of the investigated snails.

## Introduction

Molecular phylogeographic analyses can provide valuable information on specific genetic structures, genetic variation and population formation^1^. However, the population phylogeography of an organism can be affected by various factors, such as climate, geographical conditions, the ecological environment, and historical processes as well as human activities etc.^2–4^. The influence of physical barriers and environmental variations on genetic structure has been investigated in many organisms, such as *Sula leucogaster*, *Alectoris chukar* and *Adelphocoris suturalis* ^5–7^. An increasing number of researchers are now studying population genetics using cytoplasmic and nuclear genomes, due to the large amount of evolutionary information they provide^7–9^. The genetic structure of camaenids is currently poorly understood because of the difficulty of collecting specimens. In land snails, gene flow among different populations appears to be limited because of their poor migration ability^10,11^. Additionally, the morphology of the shell shows great intra-specific variation in the family Camaenidae, in terms of shell size, shape, color, spiral bands, growth lines, aperture, and the umbilicus, and these aspects may be affected by both the local environment and genetic evolution^9^. Therefore, investigation of the population genetic structure and phylogeography of camaenids will significantly enhance the current understanding of the geographic differentiation and historical biogeography of land snails.

The snail *Camaena cicatricosa* (Müller, 1774) (Stylommatophora, Camaenidae) is an important and harmful terrestrial mollusk found in southern China. It not only damages crops, leading to reductions in yield and quality, but also spreads zoonotic food-borne parasitic disease, and causes substantial damage to human and animal health^12^. The classification of this species is rather confusing, and its scientific name has been repeatedly revised. Different scholars present diverse viewpoints^13–20^. Shell morphological characteristics of *C.cicatricosa* are variable, and four synonymous names, which are *C. c. ducalis* (Ancey, 1885), *C. c. inflata* (Mllendorff, 1885), *C. c. obtecta* (Fischer, 1898) as well as *C. c. connectens* (Dautzenberg & Fischer, 1906) have been proposed. However, these subspecies are different each other in shell shape and size, openness of umbilicus, sharpness of peripheral angel, convexity of whorls and the presence of a hump beside umbilicus. These taxa previously were treated as synonyms, varieties or subspecies of *C.cicatricosa* based on only comparative shell morphology^13–20^. In 2016, the sinistral were revised and upgraded to species by Ding *et al*. according to shell morphology, anatomical as well as molecular characteristics^21^. Some studies have indicated that this snail is mainly distributed in Guangdong, Guangxi, Yunnan, Guizhou, Hunan, Hainan and Vietnam^17,22,23^. However, researchers recently clarified the phylogeny and taxonomy of sinistral camaenids, and found that this camaenid snail is distributed only in the provinces of Guangdong and Guangxi^21^. Previous studies on this snail have mainly focused on its taxonomy and mitochondrial genome^21,23^, but the phylogeography and population structure of *C. cicatricosa* have not been thoroughly understood.

In the present study, the genetic structure of this species was assessed based on mitochondrial cytochrome c oxidase subunit 1 (*COI*), *16srRNA* and nuclear internal transcribed spacer II region (ITS2) sequences. The main goals of this study were to (i) analyze the genetic diversity and structure of *C. cicatricosa* and (ii) examine the geographical pattern of its haplotypes. Genetic variation and gene flow were also evaluated, and factors influencing genetic variation were investigated.

## Results

### Genetic diversity

**Figure 1:**
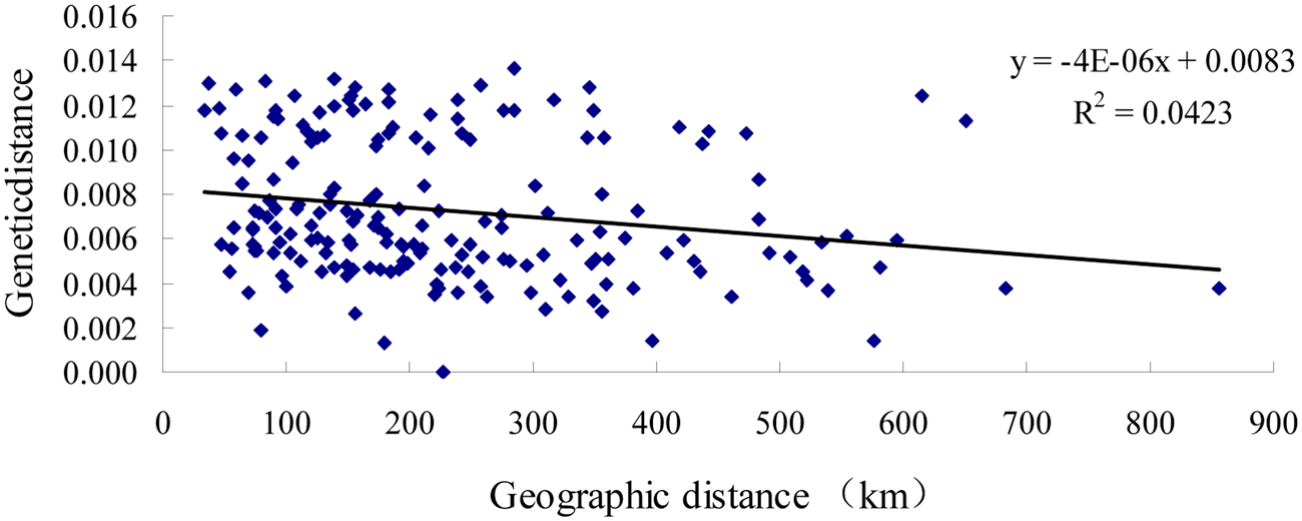
Scatter plot showing the correlation of genetic distance and geographical distance (km) based on mt DNA data.

A final combined dataset consisting 861 bp of mitochondrial gene sequences was obtained from 347 individuals, including fragments of the *COI* (559 bp) and *16srRNA* (302 bp) genes, with the following nucleotide content: 37.56% T, 13.80% C, 29.79% A and 18.85% G. A total of 46 polymorphic sites were identified including 7 singleton variables and 39 parsimony-informative sites. No insertions or deletions were detected among these fragments. The average number of nucleotide differences ranged from 0.000 to 6.725, with an average of 2.513. Haplotype diversity ranged from 0.000 to 0.848, with an average of 0.454, and nucleotide diversity from 0.000 to 0.008, with an average of 0.003 (Table S1). Thirty-nine haplotypes, including 13 shared haplotypes and 26 unique haplotypes, were derived from all of the individuals. The haplotype with the highest frequency, Hap1, was found in populations CH, GM, QY, ST, TH, ZS, NN and ZP, which accounted for 23.34%. The other frequent haplotypes included Hap13 (accounting for 8.36%), Hap3 (accounting for 7.78%), Hap5 and Hap20 (accounting for 7.2% each) (Table S2). The pairwise Fst values between all populations were significantly different (P < 0.01; P < 0.05). The maximum value between HY and GM, HJ and GM, HZ and GM, HJ and HY, HZ and HY, WZ and HY, HZ and HJ, WZ and HJ, and WZ and HZ was 1.000 uniformly, followed by values between populations ST and GM, TH and GM, ST and HY, TH and HY, ST and HJ. The smallest Fst value, between ZS and CH, was −0.048, followed by −0.015 between populations SZ and HJ (Table S3). An isolation-by-distance (IBD) test showed no significant correlation between genetic distance and geographical distance (R^2^ = 0.0423) among all of the populations, and the results indicated that gene flow was not restricted by distance (Fig. 1).

**Figure 2:**
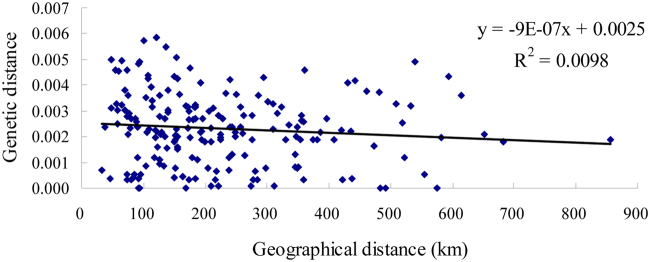
Scatter plot showing the correlation of genetic distance and geographical distance (km) based on ITS2 data.

For the ITS2 region, 347 sequences were successfully acquired, with a length of 549 bp and nucleotide content of 22.22% T, 25.93% C, 18.12% A, and 33.73% G. Forty-six polymorphic sites, including 27 singleton variable and 19 parsimony-informative sites, were obtained. The average number of nucleotide differences ranged from 0.000 to 2.788, with an average of 0.775. Haplotype diversity ranged from 0.000 to 0.824, with an average of 0.373, and nucleotide diversity from 0.000 to 0.005, with an average of 0.002 (Table S4). Twenty-one shared haplotypes and eight unique haplotypes were derived from all individuals. Hap3 was dispersed in all populations, except YD and appeared 215 times, accounting for 61.96% of the haplotypes. Other frequent haplotypes included Hap8 (accounting for 13.26%), Hap16 (accounting for 6.34%), Hap6 and Hap7 (accounting for 4.03% respectively) (Table S5). The pairwise Fst values between most populations indicated significant differences, especially between WZ and YC, YD and YC, and HJ and YC. The smallest Fst value, between HJ and CH, was −0.096 (Table S6). An IBD test showed no significant correlation between genetic distance and geographical distance (R^2^ = 0.0098) among all of the populations (Fig. 2). The results were in accordance with the mt DNA data.

### Gene flow

The pairwise Nm values between all populations were acquired based on mitochondrial gene. The largest value between CH and SH was 3.538, what followed were 2.726 between ZQ and GP, 2.087 between YD and NN, 2.044 between ZQ and ZP. The smallest Nm value was −16.917 between SZ and HJ, and the second smallest was  −5.459 between CH and ZS (Table S7). For the ITS2, five populations (LFS, NN, WZ, CH, YF) showed much higher levels of gene flow than the rest of studied populations. The largest value was 35.464, but the smallest value was merely −125.250 (Table S8).

### Phylogenetic analyses and network construction

**Figure 3:**
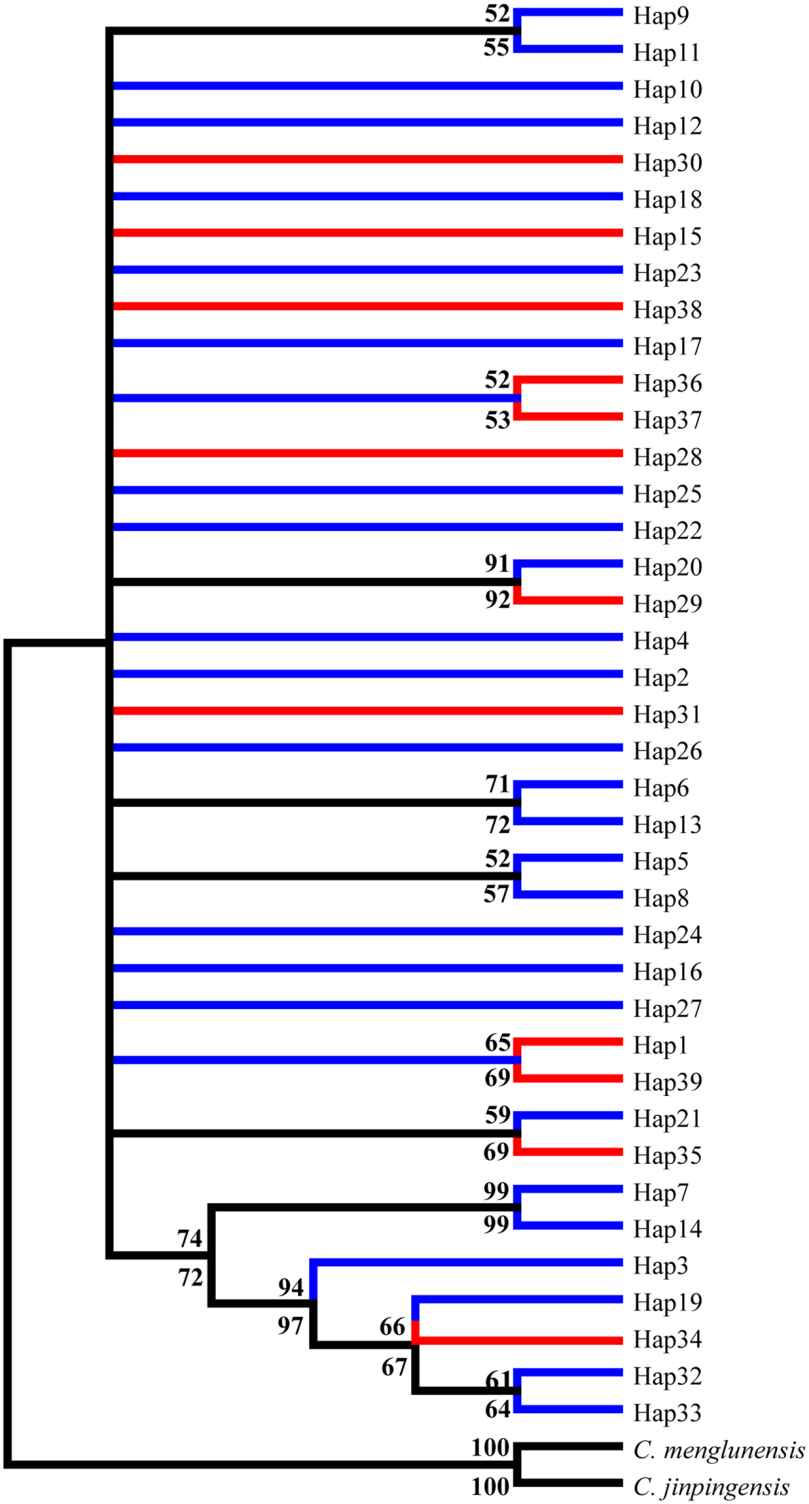
Phylogenetic tree inferred via the ML and NJ methods based on mt DNA data. Red represents the Guangxi populations; Blue represents the Guangdong populations. Numbers on and below the nodes represent ML and NJ bootstrap values respectively.

**Figure 4:**
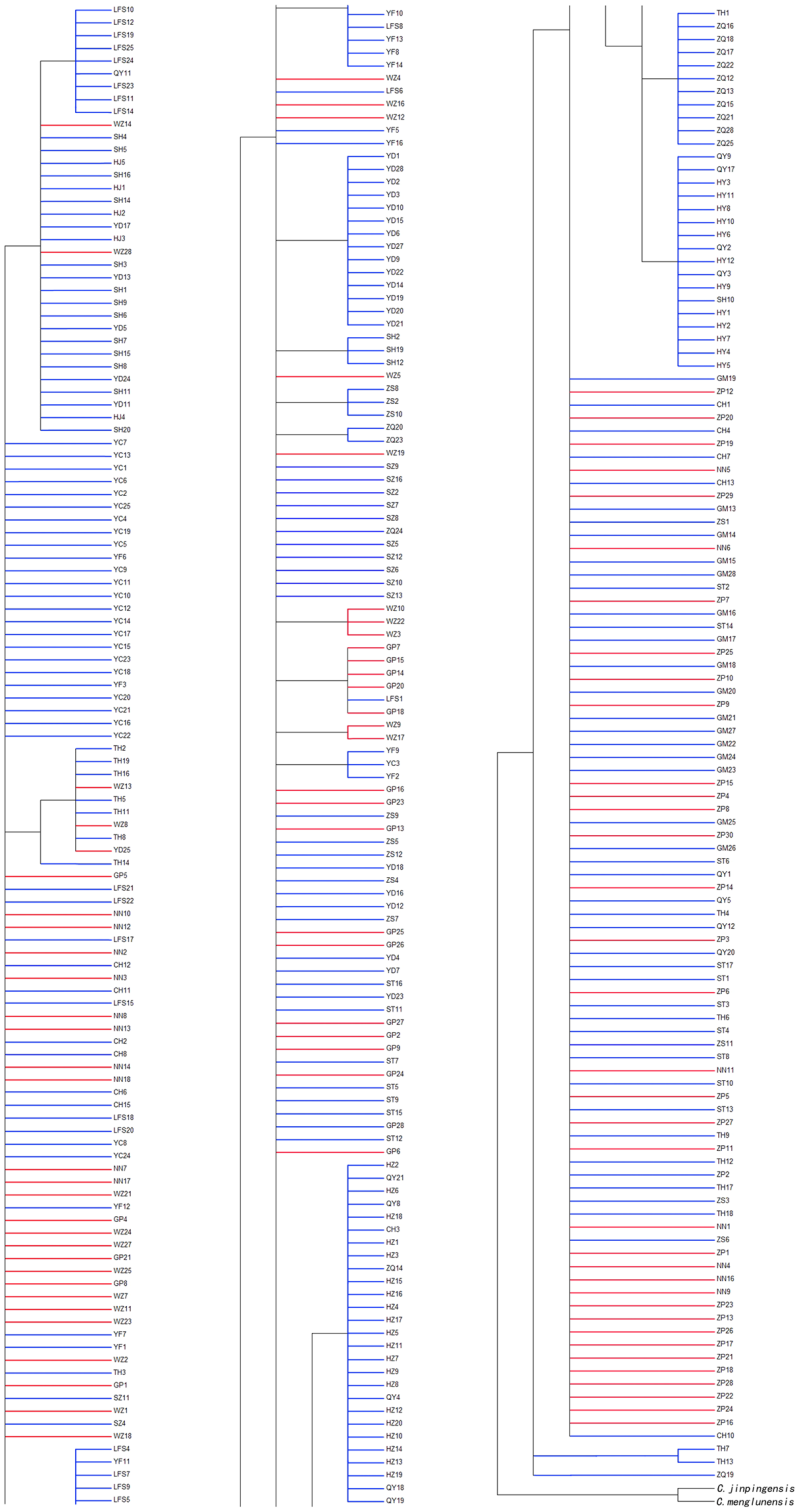
Phylogenetic tree inferred via the NJ method based on synonymous sites of the *COI* gene.

**Figure 5:**
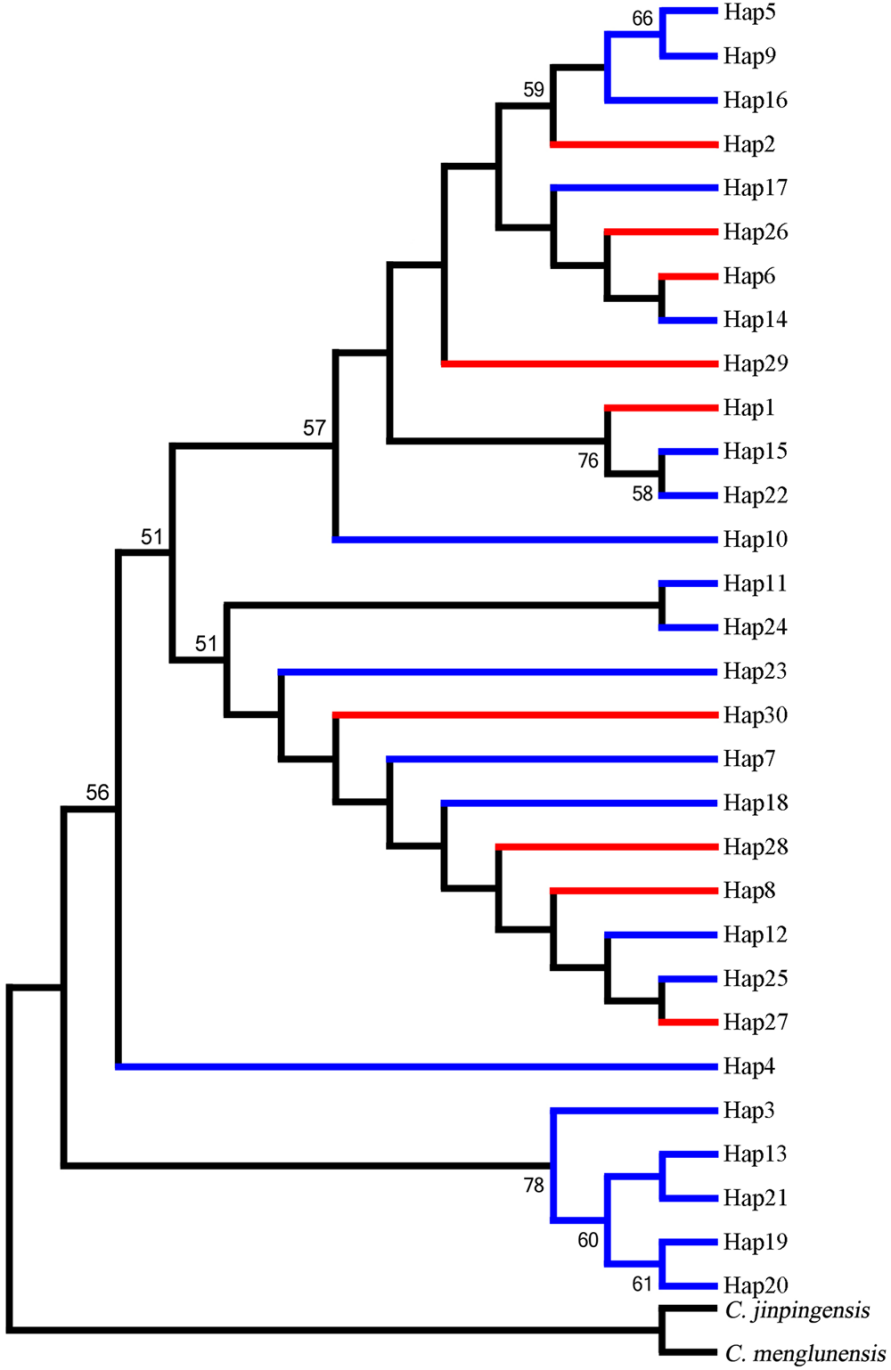
Phylogenetic tree inferred via the NJ method based on non synonymous sites of the *COI* gene.

Molecular phylogenetic analyses were conducted based on the mt DNA sequences of thirty-nine haplytypes as well as sequences from two additional specimens, *C. jinpingensis* and *C. menglunensis*, which were used as outgroup to root the tree because of their close genetic relationship. The synonymous and non synonymous sites of 347 sequences from a single *COI* gene were used to build phylogenetic trees as well. Based on the tree using the NJ method, it was observed that populations from Guangdong and Guangxi provinces were mixed together and did not present an evident structure (Figs 3, 4 and 5). In the phylogenetic tree based on synonymous sites, certain individuals from the same population showed sister relationships firstly, and then gathered together with other individuals. While in the phylogenetic tree based on non synonymous sites, all individuals except ZQ16 and ZQ20 have equal relationships.

**Figure 6:**
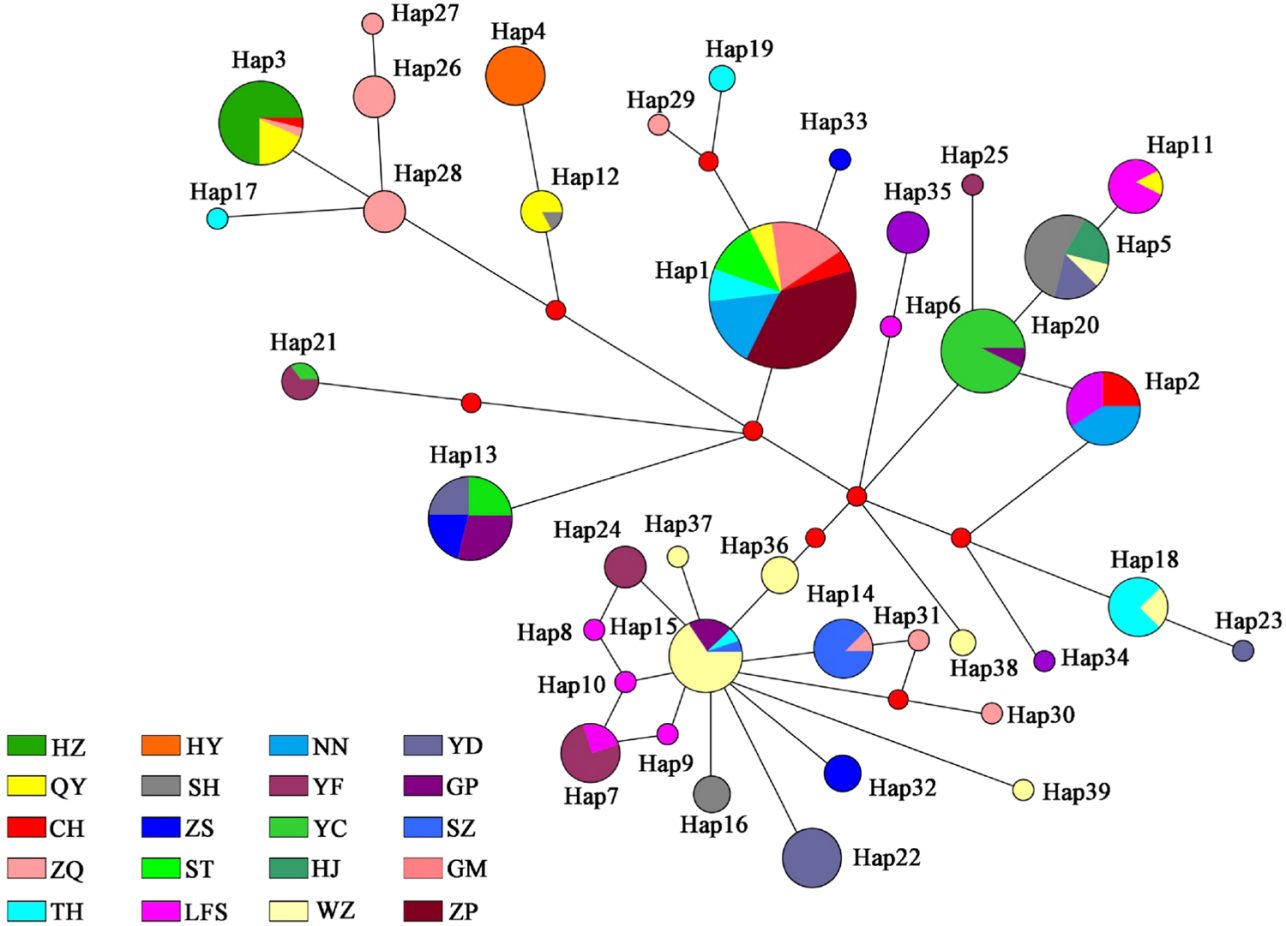
Haplotype network of *C*. *cicatricosa* based on mt DNA.

In a haplotype network, the ancient haplotype should generally be located at the center of the network, being widely distributed among populations, while, other more recent haplotypes should be located at the tips of the network. In this asteroid mitochondrial haplotype network, the most frequent haplotype (Hap1) was located at the center of the network, being distributed in populations CH, GM, QY, ST, TH, ZS, NN and ZP, including 81 individuals. Therefore, Hap1 was considered the major haplotype. The other haplotypes, including Hap3, Hap5, Hap13 and Hap20, were comprised of 27, 25, 28 and 25 individuals, respectively. One exclusive haplotype (Hap33) and two missing haplotypes had mutated from Hap1, and other haplotypes had mutated from these two missing haplotypes. Hap15, which appeared 20 times, had mutated into 10 other haplotypes and 1 missing haplotype. The star-like network suggested that Hap33, Hap29 and Hap19 were different from most other haplotypes by only few mutations (Fig. 6). Furthermore, all of the haplotypes in this network could not be divided into effective groups.

**Figure 7:**
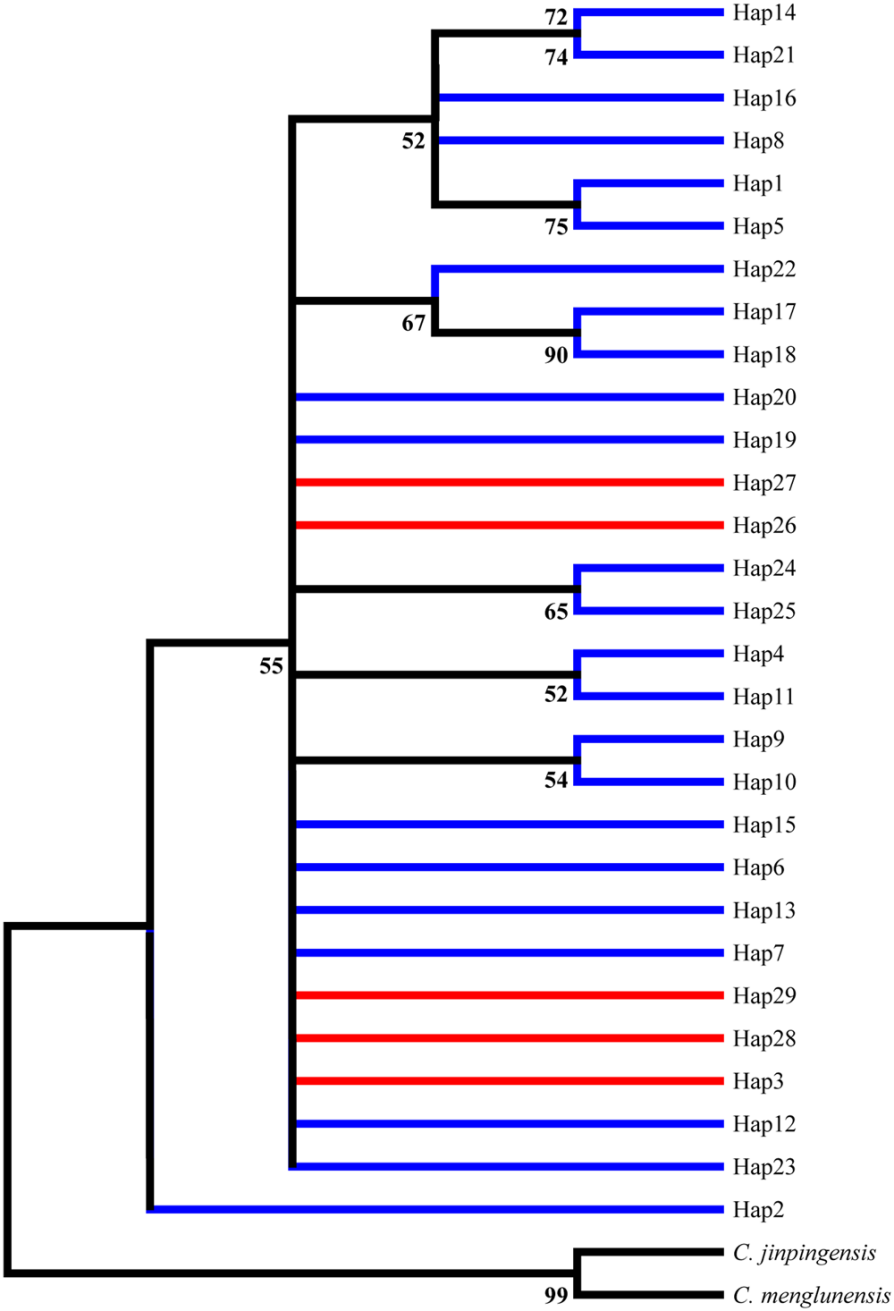
Phylogenetic tree inferred via the ML and NJ methods based on ITS2 sequences. Red represents the Guangxi populations; Blue represents the Guangdong populations. Numbers on and below the nodes represent ML and NJ bootstrap values respectively.

Molecular phylogenies were also analyzed based on ITS2 sequences from 29 haplytypes using NJ and ML methods. The snails *C. jinpingensis* and *C. menglunensis* were chosen as outgroup here as well. In these two trees, populations from Guangdong and Guangxi provinces were again mixed together and did not exhibit an evident structure (Fig. 7). This result was in accordance with the mt DNA data.

**Figure 8:**
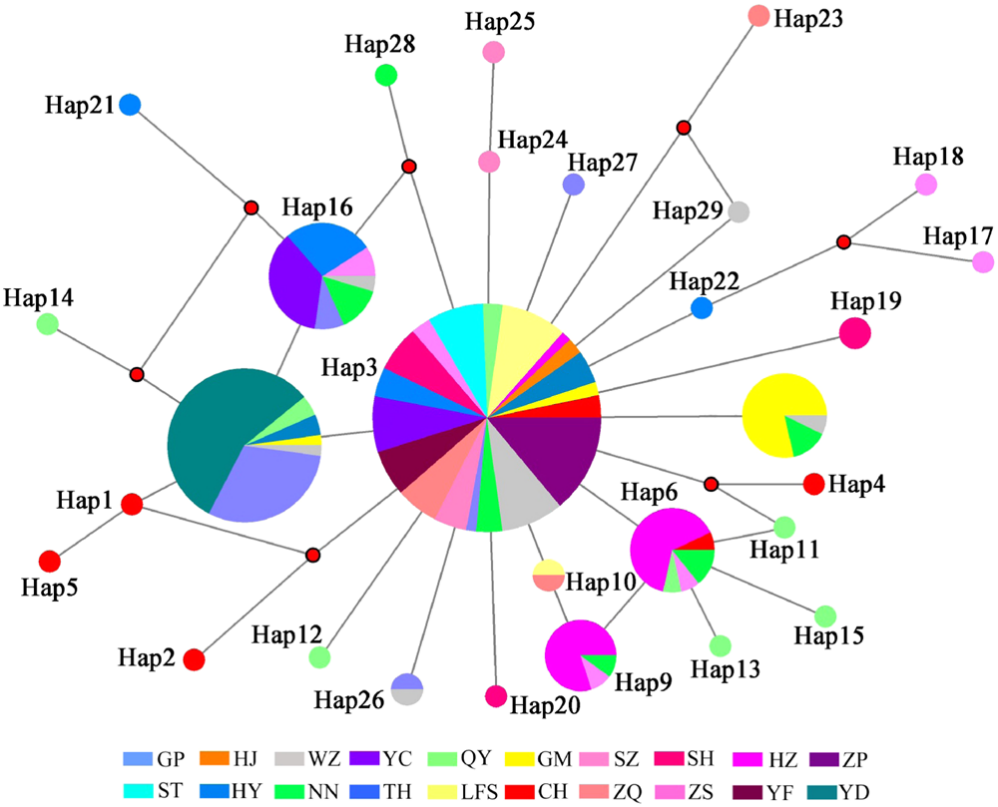
Haplotype network of *C. cicatricosa* based on ITS2 sequences.

Anther asteroid network was acquired based on ITS2 haplotypes. Hap3 was located at the center of this network and was considered the major haplotype. Two other haplotypes (Hap8 and Hap16) were also common and were distributed in additional populations. The major haplotype Hap3 appeared 215 times and generated 12 other haplotypes as well as 4 kinds of missing haplotypes. The rest of the haplotypes were likely generated from the major haplotype two or three times. Additionally, 21 exclusive haplotypes were connected with the major haplotypes through a mutation, 15 of which were located at the end of the network. The haplotype Hap3 was found in the most individuals, except the YD population from Guangdong. The populations in Guangdong included all of the shared haplotypes except Hap26. Only five haplotypes were distributed in Guangxi populations, which included only five individuals, four of which were located at the edge of the network (Fig. 8). Similar to the results based on mtDNA data, different populations of *C. cicatricosa* could not be divided into groups, indicating an inconspicuous genetic structure.

## Discussion

Genetic diversity is the basis of ecosystem diversity and species diversity, and any species has its unique gene pool or genetic organization form. Generalized genetic diversity refers to the sum of the genetic information carried by all organisms on the planet. In a narrow sense, genetic diversity refers to analyses within the species, namely genetic variation between different populations within species or different individuals in a population. Genetic diversity includes not only the level of variation, but also the distribution pattern of variation, that is, the genetic structure of population^24,25^. Up to now, there are lots of studies on species genetic diversity, including plants, insects, fish, aquatic mollusk and others^7,26–29^. However, there is no study on camaenids in this field. What factors affect the level of genetic diversity of camaenids? Which factors have great influence and which having small influence? How they impact on the weak migrating snails? Above questions are confusing. In 2012, Leffer *et al*. published a comprehensive and novel comment on the level of genetic diversity within species^30^. They pointed out that in addition to the geographic range, other complex factors such as ecological factors, life history traits and genome architecture of closely related species are expected to have discernable effects on genetic diversity, which remains incompletely understood by the present paradigm^30,31^. In the present study, we have analyzed the genetic diversity of the land snail *C. catricosa* and related influencing factors based on mitochondrial genes and ITS2 region, which is the type species of the genus *Camaena*. Although these data are limited, they can provide reference basis and valuable resources for the subsequent study.

Nucleotide transition usually appears in classification orders that show close relationship, and nucleotide transversion is evident in classification orders that show distant relationships^32,33^. In this study, nucleotide transition was the main variation observed among species of *C. cicatricosa*, which is consistent with previous research. In the genetic diversity analyses, populations GM, HZ, HJ, HY and ZP exhibited lower haplotype and nucleotide diversity (Table S1); four of these populations were distributed in Guangdong, while one was distributed in Guangxi. The landscape of Guangdong is comprised of low mountain ranges, rolling country and plains, extending from north to south. The mountains of Guangdong generally exhibit a northeast – southwest orientation. The southern border of this province faces the South China Sea, and disasters such as floods, typhoons and droughts occur often^34^. Therefore, the terrain as well as the climate may play an important role in these populations with lower genetic diversity, as observed in other organism, such as *Adelphocoris suturalis* and *Helix aspersa* ^26,27^. The ZP population in Guangxi is located in the northern area and is isolated by mountains, implying that physical barrier might also cause lower diversity level.

The values of the fixation indices between populations are among the important parameters used to measure the degree of genetic differentiation^28^. A low level of genetic differentiation exists between populations when Fst is between 0 and 0.05. Whereas, a high level of genetic differentiation exists among populations when Fst is greater than 0.15^29^. In this study, the obtained pairwise Fst values showed significant genetic differentiation among 20 populations based on the analyses of mt DNA. (Table S3). However, the phylogenetic analyses and network construction showed a lack of genetic structure. It is suggested that the high level of gene flow could result in homogeneity^39^. Almost all pairwise Fst values based on mtDNA among populations were over 0.15 (Table S3). But the data based on ITS2 sequences were not significant as those of mt DNA. It is indicated that the nuclear gene had a relatively low variability and a slow evolutionary rate compared to mt DNA^1,40^. Environment, climate and physical barriers factors may play important roles in genetic differentiation. For example, the Nanling Mountains, Pearl River, Guijiang and Xijiang River might have acted as geographical barriers^41^. On the other hand, there are many primitive forests in Guangxi including Shiwan mountain National Preserve, Yulin mountain Forest Park and so on, which served as ecological environmental and climate barriers^42^.

Gene flow is one of the main factors that are used to estimate population genetic structure. Populations with a high level of gene flow exhibit fewer genetic differentiations than populations with low gene flow^43^. When Nm > 1, high gene flow and low genetic differentiation generally exist in populations, whereas Nm < 1 indicates that populations are differentiated because of genetic drift^43^. In the present study, relatively large Nm values existed between the most populations, and values between the least populations were small (Tables S7, S8). Although this particular snail has low dispersal ability, the anthropochory, wind, water and other factors can lead to a wider distribution, especially human activities^19^. In the two examined provinces, *C. cicatricosa* is used to produce food and multi- functional medicines, and can be carried far away from its home territory^44^. Further more, trade in Guangdong province is frequent, and snails could be spread through cargo transportation too.

There are many factors that affect genetic structure and population distribution. An isolation-by-distance test showed no significant correlation between genetic distance and geographical distance among *C. cicatricosa* populations, which suggested that gene flow is not restricted by distance. Height, microclimate, host species and other factors in different locations could affect genetic structure. Recently, Huang reviewed a new hypothesis about the cause of genetic diversity in species referred to as the maximum genetic diversity (MGD) hypothesis^32^. An important novel point of the MGD hypothesis is its emphasis on the internal system or physiology of a species^45^. Because of its adaptation to mankind-disturbed environments, such as farmland and forest ecotone, this species has a large distributional range. The probability to be passively transported far distances and to establish a new population through human activities is also very high^46,47^. Besides, the snail *C. cicatricosa* is hermaphroditic creature mating with other conspecific individuals, which can laid more eggs (10–25 eggs each clutch), and has shorter gestation period (5–36 days) between the last copulation and the first egg-laying^44,48^. Organisms having higher fecundity and abundance tend to be more competitive^49^. The complex topography and geomorphology, varied physical conditions and a wide diversity of ecosystems in mountainous areas may result in allopatric and sympatric speciation^50,51^. Based on the dominance of *C. cicatricosa* in these areas, we must analyze physiological and ecological sections of this species in future research.

In the present study, the phylogenetic trees and networks of haplotypes based on the obtained datasets showed a lack of genetic structure among *C. cicatricosa* populations. These findings were confirmed by phylogenetic trees based on synonymous and non synonymous sites. Though the populations in Guangdong and Guangxi still can not be separated in the phylogenetic tree based on synonymous sites, individuals from the same population or province have closer relationships than those from different populations or provinces. The phylogenetic tree based on non synonymous sites suggested equal relationships except ZQ16 and ZQ20. That is to say the phylogenetic tree based on synonymous sites could reflect further information and make more sense. The results of this study suggested that the cause of homogeneity is the high level of gene flow, as demonstrated in other animals^39^. It is inferred that climatic changes and geographical barriers do not substantially affect the current population structure and distribution of this snail. In the future, more samples and a broader distribution of sampling locations are the necessary first step to study the genetic distribution patterns of *C. cicatricosa*. Most importantly, physiology, biology and ecology should not be overlooked.

## Methods

### Sample collection

**Figure 9:**
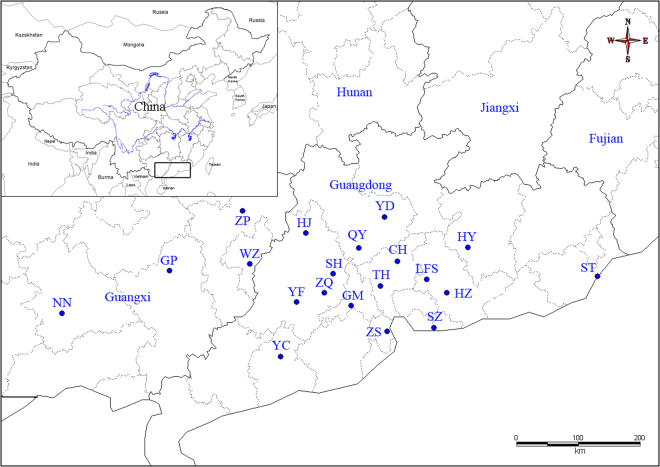
Map of sampling locations for the snail *C. cicatricosa*.

This study was based on 347 individuals collected by the authors from 20 locations in China in 2013–2015 years (Table 1, Fig. 9)^21,52,53^. The geographic coordinates were recorded using a GPS. Live adults were drowned in water for 12–24 hours and then euthanized in hot water to ensure their death. Their soft bodies were preserved in 75% or 95% ethanol and stored at −20 °C. The empty shells were cleaned, dried and preserved at room temperature. Samples were deposited in the State Key Laboratory of Molluscan Quarantine and Identification, FJIQBC.

**Table 1:** Specimen information of the snail *C. cicatricosa*.

Population code	Location, province	Coordinate	Collection time	Number of individuals
CH	Conghua, Guangdong	113°35′48.19″E; 23°31′41.16″N	2014.11.11	12
GM	Gaoming, Guangdong	112°53′02.04″E; 22°54′07.92″N	2015.03.27	16
HY	Heyuan, Guangdong	114°42′00.00″E; 23°44′05.97″N	2014.03.31	12
HJ	Huaiji, Guangdong	112°11′00.00″E; 23°56′00.00″N	2014.09.18	5
HZ	Huizhou, Guangdong	114°22′26.23″E; 23°05′05.97″N	2014.11.13	20
LFS	Luofushan, Guangdong	114°03′33.02″E; 23°16′03.67″N	2010.11.04	21
QY	Qingyuan, Guangdong	112°59′44.60″E; 23°43′16.95″N	2014.04.07	14
ST	Shantou, Guangdong	116°44′22.97″E; 23°16′59.93″N	2010.11.03	17
SZ	Shenzhen, Guangdong	114°10′12.61″E; 22°34′56.96″N	2012.07.11	12
SH	Sihui, Guangdong	112°36′12.24″E; 23°21′03.96″N	2015.03.28	17
TH	Tianhe, Guangdong	113°20′12.36″E; 23°10′31.06″N	2014.04.06	17
YC	Yangchun, Guangdong	111°47′08.00″E; 22°10′03.42″N	2014.04.01	25
YD	Yingde, Guangdong	113°24′06.05″E; 24°09′44.93″N	2014.09.17	26
YF	Yunfu, Guangdong	112°02′39.00″E; 22°56′44.78″N	2014.04.04	14
ZQ	Zhaoqing, Guangdong	112°28′11.28″E; 23°04′43.68″N	2015.03.27	15
ZS	Zhongshan, Guangdong	113°26′22.58″E; 22°31′47.38″N	2014.04.06	12
GP	Guiping, Guangxi	110°03′44.22″E; 23°23′58.87″N	2013.11.02	21
NN	Nanning, Guangxi	108°23′32.75″E; 22°47′26.69″N	2013.05.18	17
WZ	Wuzhong, Guangxi	111°18′50.66″E; 23°29′14.11″N	2013.11.03	24
ZP	Zhaoping, Guangxi	111°11′59.35″E; 24°14′58.31″N	2014.09.19	30

### DNA extraction, amplification and sequencing

Approximately 0.02–0.04 g of foot muscle tissue was used for DNA extraction. The muscle tissue was bathed in sterile water for 3–6 hours to remove residual alcohol. Total genomic DNA was isolated using the Qiagen DNeasy Blood and Tissue kit (QIAGEN), and stored at −20 °C for further use. Partial fragments of the mitochondrial *COI* and *16srRNA* genes, and the total sequence of ITS2 were amplified via PCR using the primer pairs (Table S9), reaction systems and amplification conditions listed in Table S9. The PCR products were analyzed through 1.2% agarose gel electrophoresis.

After sequencing, the raw sequences were proof-read in chromatograms and aligned into contigs using BioEdit 7.2^54^. ITS2 sequences were annotated using HMMer^55^ and the ITS2 Database^56^. The alignment of mitochondrial protein-coding genes was inferred from the amino acid alignment and examined for the presence of stop codons and other indicators. Sequence alignments were generated using ClustalW implemented in MEGA 6^57^. Sixty-seven haplotype sequences were generated and deposited in GenBank under accession numbers KU927017–KU927046 for *COI*, KX365248–KX365255 for *16srRNA* and KU958515–KU958543 for ITS2.

The nucleotide composition, mutation sites and base substitution were analyzed in MEGA 6^57^. The number of polymorphic sites (S), haplotype diversity (Hd), nucleotide diversity (Pi), average number of nucleotide differences (K) and number of haplotypes (Hap) were calculated using DnaSP 5.0^58^ or Arlequin 3.5^59^. The gene flow was estimated using DnaSP 5.0^58^ too.

The genetic differentiation among populations was further estimated based on pairwise F_ST_ values (the values of fixation indices of genetic differentiation between populations). Arlequin 3.5^59^ was employed to calculate the genetic distance matrix to test for the presence of IBD in the dataset. Google Earth (http://earth.google.com) was used to estimate the linear geographic distance (km) between the sampling locations. Significance was tested with the Mantel test employing 1000 randomizations in IBDWS 3.23^60^.

### Phylogeographic analyses and network construction

Two datasets of mitochondrial and nuclear haplotypes were analyzed through both neighbor-joining (NJ) and maximum likelihood (ML) analyses using MEGA6 with default settings. Furthermore, the synonymous and non synonymous sites of 347 sequences of the *COI* gene were employed to build phylogenetic trees. The node support values were assessed via bootstrap resampling using 1000 replicates. *C. jinpingensis* and *C. menglunensis* were employed as outgroup due to their close relationship with *C. cicatricosa*. The haplotype networks of the ITS2 and mt DNA data were constructed in Network 4.6^61^ with the median-joining algorithm.

## Electronic supplementary material

Supplementary information
