# Supplementary material for: Population genetic structure of the land snail Camaena cicatricosa (Stylommatophora, Camaenidae) in China inferred from mitochondrial genes and ITS2 sequences
Source: Sci Rep. 2017 Nov 15;7:15590. doi: 10.1038/s41598-017-15758-y (PMC5688059; doi:10.1038/s41598-017-15758-y)
Supplement: Supplementary file 1 — Supplementary information [file 41598_2017_15758_MOESM1_ESM.pdf]

Supplementary Information

Population genetic structure of the land snail *Camaena cicatricosa* (Stylommatophora, Camaenidae) in China inferred from mitochondrial genes and ITS2 sequences

Weichuan Zhou [1], Haifang Yang[2], Hongli Ding [1,3], Shanping Yang [1,3], Junhong Lin [1,3] & Pei Wang [1]*

1 Key Laboratory of Molluscan Quarantine and Identification of AQSIQ, Fujian Entry-Exit Inspection & Quarantine Bureau, Fuzhou, Fujian 350001, China.

2 National Wetland Museum of China, Hangzhou, Zhejiang 310013, China.

3 College of Plant Protection, Fujian Agriculture and Forestry University, Fuzhou, Fujian 350002, China.

* Correspondence author: wangpei601@126.com

Supplementary Table S1. Genetic diversity data based on mt DNA sequences in each population.

Population Code	N	V	Hn	Hd	Pi	K
CH	12	13	3	0.621	0.005	4.030
GM	16	0	1	0.000	0.000	0.000
HY	12	0	1	0.000	0.000	0.000
HJ	5	0	1	0.000	0.000	0.000
HZ	20	0	1	0.000	0.000	0.000
LFS	21	10	7	0.781	0.004	3.724
QY	14	17	4	0.758	0.008	6.725
ST	17	3	2	0.515	0.002	1.544
SZ	12	1	2	0.303	0.000	0.303
SH	17	13	3	0.404	0.003	2.824
TH	17	18	5	0.728	0.006	5.147
YC	25	4	2	0.080	0.001	0.320
YD	26	11	4	0.643	0.005	3.932
YF	14	10	5	0.769	0.005	3.890
ZQ	15	20	8	0.848	0.007	6.295
ZS	12	8	4	0.758	0.004	3.485
GP	21	7	4	0.662	0.003	2.724
NN	17	5	2	0.515	0.003	2.574
WZ	24	15	7	0.696	0.005	2.746
ZP	30	0	1	0.000	0.000	0.000

N: sample size; V: number of variable sites; Hn: number of halotypes; Hd: haplotype diversity; P i: nucleotide diversity; K: average number of nucleotide differences.

Supplementary Table S2. Haplotype frequency in each population based on combined mt DNA sequences.

Haplo-	Population Code																			
type	CH	GM	HY	HJ	HZ	LFS	QY	ST	SZ	SH	TH	YC	YD	YF	ZQ	ZS	GP	NN	WZ	ZP
Hap1	5	16					4	10			6					3		7		30
Hap2	6					6												10		
Hap3	1				20		5								1					
Hap4			12																	
Hap5				5						13			5						2	
Hap6						1														
Hap7						3								5						
Hap8						1														
Hap9						1														
Hap10						1														
Hap11						8	1													
Hap12							4			1										
Hap13								7					6			5	10			
Hap14									10						1					
Hap15									2		1						4		13	
Hap16										3										
Hap17											1									
Hap18											7								2	
Hap19											2									
Hap20												24		1						
Hap21												1		2						

Hap22	14						
Hap23	1						
Hap24		5					
Hap25		1					
Hap26			5				
Hap27			1				
Hap28							4
Hap29							1
Hap30							1
Hap31							1
Hap32				3			
Hap33				1			
Hap34					1		
Hap35					5		
Hap36							3
Hap37							1
Hap38							2
Hap39							1

Supplementary Table S3. Pairwise Fst values for 20 populations based on mt DNA data.

Population Code	CH	GM	HY	HJ	HZ	LFS	QY	ST	SZ	SH	TH	YC	YD	YF	ZQ	ZS	GP	NN	WZ	ZP
CH	0.000																			
GM	0.443**	0.000																		
HY	0.789**	1.000**	0.000																	
HJ	0.369**	1.000**	1.000**	0.000																
HZ	0.840**	1.000**	1.000**	1.000**	0.000															
LFS	0.191**	0.664**	0.794**	0.213*	0.829**	0.000														
QY	0.247**	0.486**	0.487**	0.448**	0.498**	0.426**	0.000													
ST	0.618**	0.981**	0.985**	0.962**	0.990**	0.506**	0.587**	0.000												
SZ	0.262**	0.738**	0.834**	-0.015*	0.880**	0.121**	0.427**	0.675**	0.000											
SH	0.066	0.327**	0.720**	0.409**	0.774**	0.291**	0.269**	0.521**	0.675**	0.000										
TH	0.398**	0.951**	0.976**	0.780**	0.982**	0.322**	0.592**	0.936**	0.347**	0.490**	0.000									
YC	0.288**	0.632**	0.733**	0.437**	0.801**	0.240**	0.436**	0.387**	0.328**	0.298**	0.510**	0.000								
YD	0.303**	0.720**	0.822**	0.531**	0.845**	0.205**	0.418**	0.364**	0.396**	0.298**	0.621**	0.201**	0.000							
YF	0.417**	0.663**	0.650**	0.567**	0.303**	0.525**	0.122*	0.655**	0.554**	0.415**	0.696**	0.535**	0.496**	0.000						
ZQ	0.122*	0.483**	0.817**	0.536**	0.860**	0.319**	0.275**	0.609**	0.391**	0.119**	0.615**	0.247**	0.279**	0.459**	0.000					
ZS	-0.048	0.554**	0.846**	0.461**	0.889**	0.218**	0.384**	0.686**	0.308**	0.135**	0.425**	0.338**	0.378**	0.537**	0.224**	0.000				
GP	0.238**	0.639**	0.816**	0.535**	0.850**	0.303**	0.369**	0.593**	0.379**	0.238**	0.545**	0.237**	0.287**	0.516**	0.084*	0.318**	0.000			
NN	0.352**	0.735**	0.838**	0.567**	0.858**	0.242**	0.490**	0.301**	0.396**	0.317**	0.613**	0.155**	0.107**	0.574**	0.319**	0.405**	0.296**	0.000		
WZ	0.561**	0.000	1.000**	1.000**	1.000**	0.737**	0.594**	0.988**	0.805**	0.428**	0.964**	0.701**	0.796**	0.748**	0.599**	0.648**	0.716**	0.793**	0.000	
ZP	0.133*	0.366**	0.779**	0.512**	0.810**	0.305**	0.309**	0.533**	0.418**	0.123*	0.592**	0.305**	0.223**	0.459**	0.109**	0.221**	0.273**	0.323**	0.468**	0.000

*:p<0.05； **P<0.01

Supplementary Table S4. Genetic diversity data based on ITS2 sequences in each population.

Population Code	N	V	Hn	Hd	Pi	K
CH	12	13	6	0.682	0.005	2.545
GM	16	2	3	0.492	0.001	0.583
HY	12	1	2	0.303	0.001	0.303
HJ	5	0	1	0.000	0.000	0.000
HZ	20	2	3	0.647	0.002	0.774
LFS	21	1	2	0.095	0.000	0.095
QY	14	11	8	0.824	0.004	2.088
ST	17	0	1	0.000	0.000	0.000
SZ	12	11	6	0.758	0.005	2.788
SH	17	2	3	0.324	0.001	0.338
TH	17	6	4	0.625	0.003	1.500
YC	25	2	2	0.453	0.002	0.907
YD	26	0	1	0.000	0.000	0.000
YF	14	0	1	0.000	0.000	0.000
ZQ	15	3	3	0.257	0.001	0.400
ZS	12	2	3	0.318	0.001	0.470
GP	21	4	5	0.548	0.001	0.752
NN	17	6	6	0.757	0.003	1.456
WZ	24	5	6	0.380	0.001	0.493
ZP	30	0	1	0.000	0.000	0.000

N: sample size; V: number of variable sites; Hn: number of halorypes; Hd: haplotype diversity; Pi: nucleotide diversity; K: average number of nucleotide differences.

Supplementary Table S5. Haplotype frequency based on ITS2 sequences in each population.

Haplotype	Population Code																			
	CH	GM	HY	HJ	HZ	LFS	QY	ST	SZ	SH	TH	YC	YD	YF	ZQ	ZS	GP	NN	WZ	ZP
Hap1	1																			
Hap2	1																			
Hap3	7	4	10	5	3	20	6	17	6	14	9	17		14	13	10	3	8	19	30
Hap4	1																			
Hap5	1																			
Hap6	1				9		1		1									2		
Hap7		11																2	1	
Hap8		1	2				2						26				14		1	
Hap9					8				1									1		
Hap10						1									1					
Hap11							1													
Hap12							1													
Hap13							1													
Hap14							1													
Hap15							1													
Hap16									2		6	8					2	3	1	
Hap17									1											
Hap18									1											
Hap19										2										
Hap20										1										
Hap21											1									
Hap22											1									
Hap23															1					
Hap24																1				
Hap25																1				
Hap26																	1		1	
Hap27																	1			
Hap28																		1		
Hap29																			1	

Supplementary Table S6. Pairwise Fst values for 20 populations based on ITS2 data.

Population Code	CH	GM	HY	HJ	HZ	LFS	QY	ST	SZ	SH	TH	YC	YD	YF	ZQ	ZS	GP	NN	WZ	ZP
CH	0.000																			
GM	0.374	0.000																		
HY	-0.012	0.553**	0.000																	
HJ	-0.120	0.507*	-0.017	0.000																
HZ	0.358**	0.665**	0.591**	0.556**	0.000															
LFS	0.018	0.649**	0.100	-0.105	0.660**	0.000														
QY	0.017	0.393**	0.024	-0.028	0.271**	0.123**	0.000													
ST	0.043	0.359**	0.037	-0.035	0.298**	0.121**	0.010	0.000												
SZ	0.037	0.577**	0.062	-0.069	0.601**	0.041	0.094**	0.094**	0.000											
SH	0.141	0.476**	0.146*	0.143	0.514**	0.307**	0.110*	0.059	0.256**	0.000										
TH	0.122	0.524**	0.101	0.126	0.552**	0.254**	0.111*	0.070	0.216**	-0.023	0.000									
YC	0.563**	0.820**	0.882**	1.000**	0.847**	0.959**	0.495**	0.487**	0.884**	0.456**	0.549**	0.000								
YD	-0.022	0.622**	0.109	0.000	0.650**	-0.021	0.087**	0.086**	0.028	0.267**	0.226*	1.000**	0.000							
YF	0.014	0.555**	0.037	-0.099	0.566**	-0.015	0.078**	0.071*	0.020	0.236**	0.198**	0.873**	-0.004	0.000						
ZQ	0.019	0.536**	0.071	-0.042	0.568**	0.088	0.071*	0.066*	0.057	0.223*	0.198*	0.876**	0.078	0.034	0.000					
ZS	0.054	0.324**	0.035	-0.023	0.338**	0.108**	0.015	0.004	0.089**	0.051	0.032	0.549**	0.082*	0.068	0.071*	0.000				
GP	0.310**	0.613**	0.361**	0.456**	0.653**	0.579**	0.222**	0.221**	0.507**	0.142*	0.203**	0.142**	0.558**	0.486**	0.474**	0.255**	0.000			
NN	0.037	0.513**	-0.032	-0.096	0.565**	0.007	0.074**	0.078*	0.018	0.186**	0.127*	0.751**	-0.013	-0.003	0.029	0.039	0.403**	0.000		
WZ	0.033	0.725**	0.216	0.000	0.739**	0.018	0.178**	0.186**	0.091*	0.383**	0.316**	1.000**	0.000	0.051	0.175	0.162**	0.658**	0.026*	0.000	
ZP	-0.009	0.647**	0.133	0.000	0.672**	-0.010	0.108**	0.109**	0.043	0.294**	0.246*	1.000**	0.000	0.009	0.100	0.101**	0.581**	-0.002	0.012**	0.000

*:p<0.05； **P<0.01

Supplementary Table S7. Pairwise Nm values for 20 populations based on mt DNA data.

Population Code	CH	GM	HY	HJ	HZ	LFS	QY	ST	SZ	SH	TH	YC	YD	YF	ZQ	ZS	GP	NN	WZ	ZP
CH	-																			
GM	0.315	-																		
HY	0.067	0.000	-																	
HJ	0.428	0.000	0.000	-																
HZ	0.046	0.000	0.000	0.000	-															
LFS	1.059	0.127	0.065	0.924	0.052	-														
QY	0.762	0.265	0.264	0.308	0.252	0.337	-													
ST	0.155	0.005	0.004	0.010	0.003	0.244	0.176	-												
SZ	0.704	0.089	0.050	-16.917	0.034	1.816	0.336	0.121	-											
SH	3.538	0.515	0.097	0.361	0.073	0.609	0.680	0.230	0.121	-										
TH	0.378	0.013	0.006	0.071	0.005	0.527	0.173	0.017	0.471	0.260	-									
YC	0.618	0.146	0.091	0.322	0.062	0.792	0.324	0.396	0.512	0.589	0.240	-								
YD	0.575	0.097	0.054	0.221	0.046	0.970	0.348	0.437	0.382	0.589	0.153	0.994	-							
YF	0.350	0.127	0.135	0.191	0.575	0.226	1.799	0.132	0.202	0.353	0.109	0.218	0.254	-						
ZQ	1.799	0.268	0.056	0.217	0.041	0.534	0.659	0.161	0.390	1.851	0.157	0.762	0.646	0.295	-					
ZS	-5.459	0.202	0.046	0.293	0.031	0.897	0.401	0.115	0.562	1.602	0.338	0.490	0.412	0.216	0.866	-				
GP	0.801	0.141	0.057	0.212	0.044	0.575	0.428	0.172	0.410	0.801	0.209	0.805	0.621	0.235	2.726	0.536	-			
NN	0.460	0.090	0.049	0.191	0.042	0.783	0.26	0.581	0.382	0.539	0.158	1.363	2.087	0.186	0.534	0.368	0.595	-		
WZ	0.196	0.000	0.000	0.000	0.000	0.089	0.171	0.003	0.061	0.334	0.010	0.107	0.064	0.084	0.168	0.136	0.099	0.066	-	
ZP	1.630	0.433	0.071	0.239	0.059	0.570	0.559	0.219	0.348	1.783	0.173	0.570	0.871	0.295	2.044	0.881	0.666	0.524	0.284	-

Supplementary Table S8. Pairwise Nm values for 20 populations based on ITS2 data.

Population Code	CH	GM	HY	HJ	HZ	LFS	QY	ST	SZ	SH	TH	YC	YD	YF	ZQ	ZS	GP	NN	WZ	ZP
CH	-																			
GM	0.418	-																		
HY	-21.083	0.202	-																	
HJ	-2.333	0.243	-14.956	-																
HZ	0.448	0.126	0.173	0.200	-															
LFS	13.639	0.135	2.250	-2.631	0.129	-														
QY	14.456	0.386	10.167	-9.179	0.673	1.783	-													
ST	5.564	0.446	6.507	-7.393	0.589	1.816	24.750	-												
SZ	6.507	0.183	3.782	-3.873	0.166	5.848	2.410	2.410	-											
SH	1.523	0.275	1.462	1.498	0.236	0.564	2.023	3.987	0.727	-										
TH	1.799	0.227	2.225	1.734	0.203	0.734	2.002	3.321	0.907	-11.120	-									
YC	0.194	0.055	0.033	0.000	0.045	0.011	0.255	0.263	0.033	0.298	0.205	-								
YD	-11.614	0.152	2.044	0.000	0.135	-12.155	2.624	2.657	8.679	0.686	0.856	0.000	-							
YF	17.607	0.200	6.507	-2.775	0.192	-16.917	2.955	3.271	12.25	0.809	1.013	0.036	-62.750	-						
ZQ	12.908	0.216	3.271	-6.202	0.190	2.591	3.271	3.538	4.136	0.871	1.013	0.035	2.955	7.103	-					
ZS	4.380	0.522	6.893	-11.12	0.490	2.065	16.417	62.25	2.559	4.652	7.563	0.205	2.799	3.426	3.271	-				
GP	0.556	0.158	0.443	0.298	0.133	0.182	0.876	0.881	0.243	1.511	0.982	1.511	0.198	0.264	0.277	0.73	-			
NN	6.507	0.237	-8.063	-2.854	0.192	35.464	3.128	2.955	13.639	1.094	1.719	0.083	-19.481	-83.583	8.371	6.16	0.370	-		
WZ	7.326	0.095	0.907	0.000	0.088	13.639	1.154	1.094	2.497	0.403	0.541	0.000	0.000	4.652	1.179	1.293	0.130	9.365	-	
ZP	-28.028	0.136	1.630	0.000	0.122	-25.250	2.065	2.044	5.564	0.600	0.766	0.000	0.000	27.528	2.250	2.225	0.180	-125.250	20.583	-

Supplementary Table S9. PCR Primer pairs and conditions used in the analyses.

**Gene**	***COI***
Primer pairs (5’-3’)	LCO:GGTCAACAAATCATAAAGATATTGG<br>HCO:TAAACTTCAGGGTGACCAAAAAATCA
Reaction systems	25ul Taq PCR MasterMix×2; 1ul each primer; 2ul DNA; 16ul ddH2O
Cycling conditions	94°C: 30s; 94°C: 10s, 45°C: 50s, 72°C: 1min, 40 cycles; 72°C: 10min.
Reference	52
**Gene**	***16srRNA***
Primer pairs (5’-3’)	16SAR:CGCCTGTTTATCAAAAACAT<br>16SBR:CCGGTCTGAACTCAGATCACGT
Reaction systems	25ul Taq PCR MasterMix×2; 1ul each primer; 2ul DNA; 16ul ddH2O
Cycling conditions	94°C: 30s; 94°C: 10s, 45°C: 50s, 72°C: 1min50s, 40 cycles; 72°C: 10min.
Reference	53
**Sequence**	**ITS2**
Primer pairs (5’-3’)	FYIT2:CATCGACATCTTGAACGCACAT<br>RYIT2:TCCCAAACAACCCGACTCCT
Reaction systems	25ul Taq PCR MasterMix×2; 1ul each primer; 2ul DNA; 16ul ddH2O
Cycling conditions	94°C: 30s; 94°C: 10s, 55°C: 30s, 72°C: 1min30s, 40 cycles; 72°C: 10min.
Reference	21
